# ATAC2GRN: optimized ATAC-seq and DNase1-seq pipelines for rapid and accurate genome regulatory network inference

**DOI:** 10.1186/s12864-018-4943-z

**Published:** 2018-07-31

**Authors:** Thomas J. F. Pranzatelli, Drew G. Michael, John A. Chiorini

**Affiliations:** National Institute of Dental and Craniofacial Research, National Institutes of Health, 10 Center Drive, Bethesda, MD 20816 USA

**Keywords:** DNA footprinting, Pipeline, ATAC-seq, DNase1-seq, Regulation, Optimization

## Abstract

**Background:**

Chromatin accessibility profiling assays such as ATAC-seq and DNase1-seq offer the opportunity to rapidly characterize the regulatory state of the genome at a single nucleotide resolution. Optimization of molecular protocols has enabled the molecular biologist to produce next-generation sequencing libraries in several hours, leaving the analysis of sequencing data as the primary obstacle to wide-scale deployment of accessibility profiling assays. To address this obstacle we have developed an optimized and efficient pipeline for the analysis of ATAC-seq and DNase1-seq data.

**Results:**

We executed a multi-dimensional grid-search on the NIH Biowulf supercomputing cluster to assess the impact of parameter selection on biological reproducibility and ChIP-seq recovery by analyzing 4560 pipeline configurations. Our analysis improved ChIP-seq recovery by 15% for ATAC-seq and 3% for DNase1-seq and determined that PCR duplicate removal improves biological reproducibility by 36% without significant costs in footprinting transcription factors. Our analyses of down sampled reads identified a point of diminishing returns for increased library sequencing depth, with 95% of the ChIP-seq data of a 200 million read footprinting library recovered by 160 million reads.

**Conclusions:**

We present optimized ATAC-seq and DNase-seq pipelines in both Snakemake and bash formats as well as optimal sequencing depths for ATAC-seq and DNase-seq projects. The optimized ATAC-seq and DNase1-seq analysis pipelines, parameters, and ground-truth ChIP-seq datasets have been made available for deployment and future algorithmic profiling.

**Electronic supplementary material:**

The online version of this article (10.1186/s12864-018-4943-z) contains supplementary material, which is available to authorized users.

## Background

Regulatory control of RNA expression is a key component of the central dogma of molecular biology. Under the central dogma, protein and RNA products regulate RNA expression. Modules of expression compose expression states associated with specialized biological activities. The information contained within a genome is expressed in a temporally and spatially coordinated program of ever increasing differentiation. This program starts with the embryonic stem cell and grows progressively more specialized. Control over RNA expression is predominantly achieved through the cooperative behavior of multiple proteins that bind DNA called transcription factors (TF) which modify chromatin and recruit RNA polymerase II (RNAP) prior to transcriptional elongation and mRNA expression [1].

Higher order control over RNA expression has been postulated to exist in terms of an ‘epigenetic landscape’ [2] in which expression of transcription factors at specific developmental states and lineages restricts the possible set of expression modules that can be activated. Thus, cells are limited to a stable expression state vector that is associated with a given lineage and cell type. Multiple RNA profiling technologies have been developed to rapidly and comprehensively identify stable transcriptional states including DNA microarrays and, recently, RNA-seq via Next-Generation Sequencing (NGS) [3]. While RNA profiling technologies may comprehensively identify the RNA stable state associated with a given cell type (or cell in the case of single cell RNA-seq), they do not provide information on the chromatin accessibility patterns and transcription factor binding events that produce this stable state.

Chromatin accessibility profiling was developed to interrogate the genomic circumstances that drives a given expression state [4]. These techniques profile the accessibility of the DNA to enzymatic digestion or transposition, that is regions of the genome occupied by protein complexes (e.g., nucleosomes, transcription factor complexes). Commonly used techniques for chromatin accessibility profiling include DNase1 hypersensitivity profiling (DNase1-seq) [5] and the Assay for Transposase-Accessible Chromatin using Sequencing (ATAC-seq) [6]. Briefly, nuclei are extracted from a sample and treated with concentrations of enzyme that result in fewer cuts/insertions in regions of high nucleosome occupancy. At lower read depths (e.g., 40–60 million reads), the nucleosome occupancy of the genome can be effectively identified. At higher read depths (e.g., > 200 million reads), subtly lower rates of enzyme activity reveal genomic intervals of transcription factor bound DNA, commonly described as ‘footprints’ [7]. These footprints describe a subset of the protein bound genomic DNA and enable genome-wide reconstruction of the cellular gene regulatory network from a single experiment. Importantly, transcription factor binding kinetics and DNA-binding domain types impact the presence and detectability of this footprint, with some TFs leaving well-defined footprints and others leaving poorly-defined footprints [8]. Despite these limitations, DNase1 and ATAC-seq-based genomic footprinting represent a rapid and cost-effective means to build a model of cellular gene regulation that can later be supplemented with traditional gene regulatory network inference methods [9–12].

The process of cellular gene regulatory network (GRN) reconstruction via TF footprinting is computationally intensive and requires multiple data transformations, each of which have multiple possible parameters that may or may not significantly impact the quality and accuracy of the network generated. To assess the impact of parameter selection on network generation, we executed a computational grid search across eight parameter arguments and five different pipeline steps, with a total of 4560 different pipelines evaluated. The process of evaluating 4560 different pipelines collectively utilized 72,960 CPUs with 3.75 GB RAM each. The mean total time that each pipeline took was 2 h, and running all pipelines on the Biowulf supercomputing system consumed more than 100,000 CPU hours (http://hpc.nih.gov) for each run. The results from each of the 4560 argument combinations was then assessed against ‘ground truth’ ENCODE chromatin immunoprecipitation (ChIP)-seq results describing TF binding within the GM12878 lymphoblastoid cell line for both DNase1 hypersensitivity and ATAC-seq chromatin accessibility profiling [13]. We present the results of this grid search to provide investigators insight to the impact of parameters on pipeline function. Additionally, we provide optimized Snakemake and bash pipelines with pre-configured algorithmic parameters capable of maximizing biological reproducibility and network accuracy for DNase1 hypersensitivity profiling and ATAC-seq [14].

## Results

### Metrics used

**Fig. 1:**
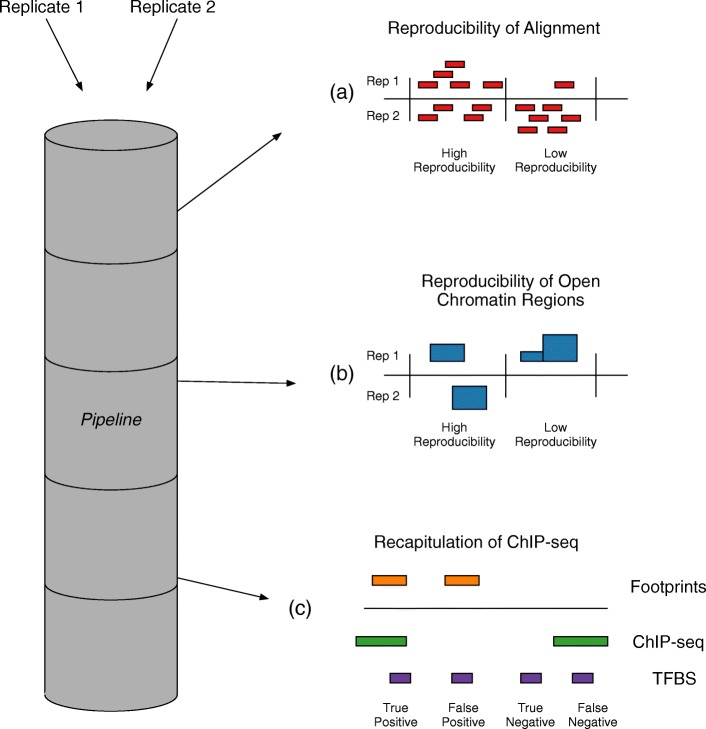
Visualization of pipeline metrics **(a)** Alignment reproducibility is measured as the correlation between two replicates in the number of reads that fall inside 10 kb genomic bins. Bins with similar read densities in each replicate indicate higher reproducibility; bins with an imbalance signify lower reproducibility. **b** Open chromatin reproducibility is measured by assessing average peak height across all bases in the window compared between replicates. Collections of bins in which the average peak height is similar between replicates result in high reproducibility scores. **c** To measure biological significance, known transcription factor binding information from ChIP-seq is compared to predicted protein binding footprints at each site that is a match to a protein’s binding site motif. If the footprint prediction lines up with known ChIP-seq data, the binding motif is a true positive protein binding site; if the footprint prediction does not line up with ChIP-seq data, the binding motif is a false positive. Likewise, if a binding motif lines up with ChIP-seq but has no footprint, it is a false negative, and if the binding motif lines up with neither ChIP-seq nor a footprinting prediction it is a true negative

Four metrics were calculated: alignment reproducibility, open chromatin reproducibility, TF footprint reproducibility, and recapitulation of known TF ChIP-seq data (Fig. 1).

### Footprints increase linearly with reads – ChIP-seq recovery yields diminishing returns with read depths greater than 60 million readz

**Fig. 2:**
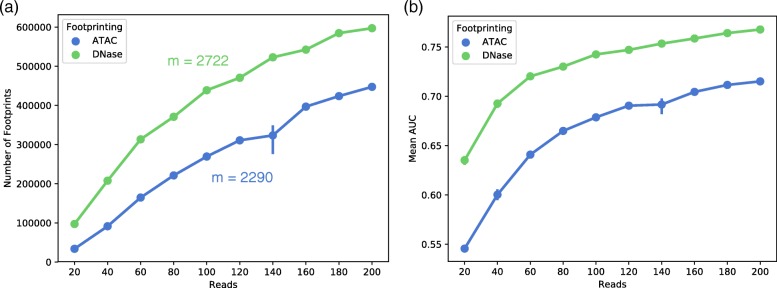
The impact of library read depth on number of footprints detected and ChIP-seq recovery as measured by AUC. The number of footprints and biological information of those footprints was calculated in a single downsampled library. Error bars depict the 95% confidence interval around mean values at each downsampling (*n* = 3). **a** Number of footprints were measured against read depths at each downsampling. The number of detected footprints increases linearly with read depth. **b** Mean AUC was measured as a function of downsampled read depth. The increase in AUC with additional reads exhibits a diminishing return per read as read depths increase beyond 100 million reads

ATAC-seq and DNase1-seq reads were ‘downsampled’ (reduced in sample size) to investigate the relationship between read depth, number of footprints detected, and ChIP-seq recovery. Reads were sampled from 20 to 200 million single-end reads; the number of open chromatin regions, the number of footprints, and the ChIP-seq AUC was calculated at each sampling (Fig. 2). Our analysis indicated that the number of detected TF footprints scaled with read depth according to a slope of 2722 footprints per additional million reads for DNase1 and 2290 footprints per additional million reads for ATAC (Fig. 2a). Inspection of the downsampled data indicated that a reduction of read depth explained 73 and 98% of the variance in ATAC-seq and Dnase1-seq footprint number respectively. The number of footprints detected was also linearly related to the number of peaks, indicating that downsampling primarily impacts the ability of the system to detect footprints by restricting the ability of the system to detect a peak. ROC AUC (area under the repeater operating characteristic curve), a measure of a predictor’s ability to predict true positives, increased non-linearly as read depths increased (Fig. 2b). Put another way, accurate footprint recovery of TF binding as measured by ChIP continued to increase but at a lower rate at higher read depths. Analysis of ChIP recovery indicates diminishing return in the significance of the footprints produced with at read depths greater than 60 million reads. ChIP-seq recovery as measured by ROC AUC continued to improve as the number of reads accumulated. Fitting the mean-AUC-to-reads relationship to a power function gave a rough expectation of the number of reads necessary to achieve a given mean AUC. To predict the number of reads required to achieve an AUC of 0.95, we fit a function to the DNase1-seq and ATAC-seq data. This analysis indicated that 2.85 billion reads of DNase1-seq data to reach a 0.95 mean AUC, while the function for ATAC-seq data projected a total of 2.15 billion reads to reach the same AUC. Wellington’s performance using the default arguments was particularly poor leading to extreme improvements by Wellington in mean AUC over random of 10,355 and 1013% for DNase- and ATAC-seq, respectively.

### PCR duplicate removal

**Fig. 3:**
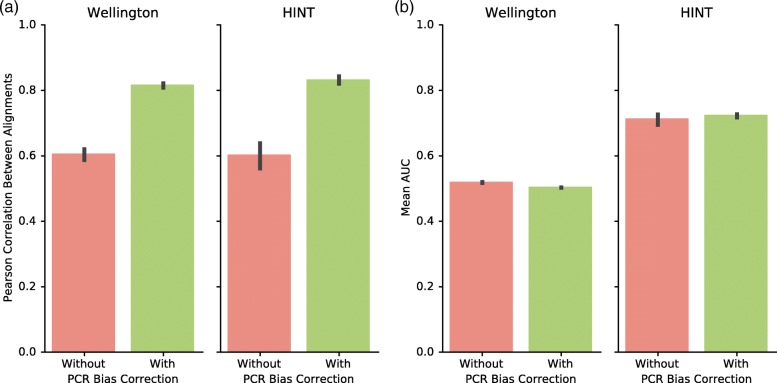
PCR duplicate removal improves biological reproducibility. Pearson correlation representing reproducibility between replicates and mean AUC representing biological information plotted for each footprinting algorithm with and without PCR duplicate removal. Error bars represent 95% confidence interval. **a** Across all parameter combinations, Pearson correlation coefficients were plotted with and without PCR duplicate removal for each footprinting algorithm. PCR duplicate removal improves biological reproducibility in both HINT and Wellington driven pipelines. **b** Across all parameter combinations, mean AUC was plotted with and without PCR duplicate removal for each footprinting algorithm. ChIP-seq recovery does not significantly improve in either algorithm with PCR bias correction. AUC-optimized pipelines for HINT use PCR bias correction, while AUC-optimized pipelines for Wellington do not

As part of the procedure for ATAC-seq and DNase-seq, the enzymatically-digested genomic fragments are PCR amplified. This process of PCR amplification can introduce bias into the composition of the library via stochastic ‘PCR jackpotting’ in which randomly selected genomic intervals are amplified by the DNA polymerase [15]. We evaluated the impact of PCR duplicate removal by assessing footprinting pipelines that included combinations of input data with no PCR duplicate removal and those with PCR duplicate removal, using SAMtools and Picard to transform and index incoming alignment files (Fig. 3).

When PCR duplication removal was introduced, the reproducibility of footprinting data alignments measured as Pearson correlation between two replicates’ genomic bins increased from 0.6 to 0.82 (Fig. 3a). Introducing PCR duplication removal did not significantly affect ChIP-seq recovery for the footprints produced from Wellington or HINT. Although the pipelines with highest AUCs for HINT incorporated PCR duplicate removal, this improvement was not statistically significant (Fig. 3b). Therefore, PCR duplicate removal did not improve the ability of Wellington TF footprinting to recapitulate ChIP-seq data. Overall, PCR duplicate removal improves biological reproducibility without significant impacts on the detection of transcription factor binding events.

### Open chromatin regions

**Fig. 4:**
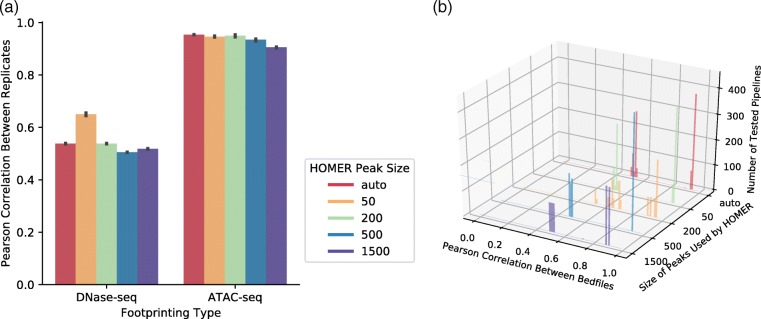
Impact of HOMER peak parameters on reproducibility. Assessment of the impact of HOMER peak sizes on DNase1-seq and ATAC-seq isogenic reproducibility. **a** Pearson correlation coefficients for average peak height in genomic bins was measured for each peak size parameter for all parameter combinations, for each data type. **b** Distributions of Pearson correlation coefficients between replicates are plotted for each peak size. Smaller peak sizes result in higher reproducibility of the openness of 10 kb genomic windows between replicates. Results are shown in one plot for both DNase1-seq and ATAC-seq. Note that ATAC-seq replicates were produced within one lab whereas the DNase1-seq libraries were from two ENCODE groups

When calling open chromatin regions, peak detection algorithms scan across the genome, finding uniformly-sized bins that contain an enriched number of aligned reads. Following peak detection, overlapping bins are stitched together to form open chromatin regions of high read density. Our pipelines utilized the HOMER software suite to identify open chromatin regions. Two arguments to HOMER were assessed across the grid search: *i*) peak size, controlling the size of the bins; and *ii*) minDist, controlling the minimum distance allowed between two peaks. Analysis of grid search results indicates that peak size altered the biological reproducibility of detected open chromatin regions (Fig. 4a, b).

Assessment of replicate correlation scores indicates that smaller peak sizes show higher mean reproducibility scores for both DNase- and ATAC-seq but also limited the area in which footprints could be called. This smaller search space seems to explain the lower AUC seen in lower peak sizes. To confirm that open chromatin regions extended beyond where reads aligned within known regulatory regions, a subset of promoters were visually inspected. Visual inspection of BAM files in IGV indicated that the use of progressively larger peak sizes results in errors of open chromatin region calls in genomic intervals associated with large numbers of aligned reads (Additional file 4: Figure S3a). Thus, a tradeoff exists between expanding footprinting across cis-regulatory regions and containing footprinting within truly open chromatin. The pipelines with the highest recovery of ChIP-seq used peak sizes of 200–500. Altering the minimum distance between peaks had no effect on reproducibility or footprint value (Additional file 4: Figure S3C-E).

### Reproducibility vs. transcription factor ChIP recovery

**Fig. 5:**
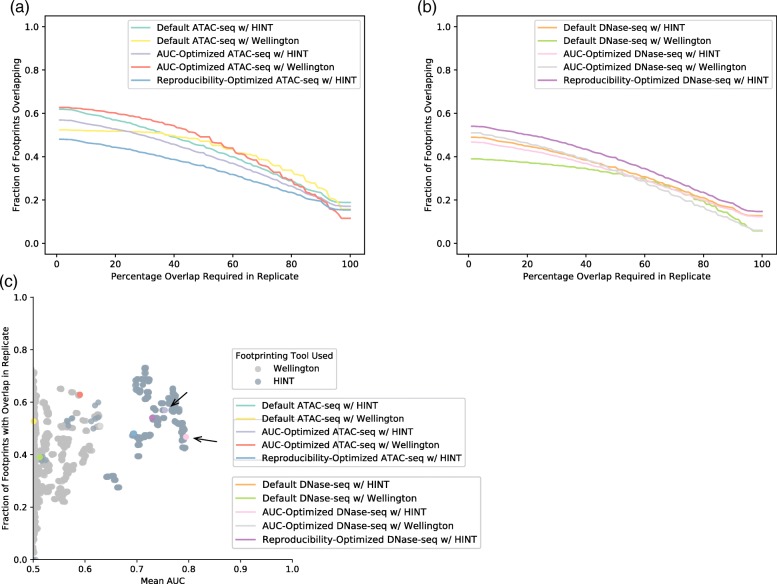
Assessment of transcription factor footprint reproducibility across biological replicates for ATAC-seq and DNase1-seq. **a** ATAC-seq footprint biological reproducibility was investigated by overlapping the footprints of replicates and measuring the number of footprints with an overlapping replicate footprint. The percentage of overlap required between the two replicates was increased and the proportion of still-overlapping footprints plotted. For ATAC-seq data, the pipeline optimized to reproduce intermediate alignment and OCR files does not produce reproducible footprints as well as AUC-optimized pipelines. **b** Dnase1-seq footprint biological reproducibility was investigated by overlapping the footprints of replicates and measuring the number of footprints with an overlapping replicate footprint. The percentage of overlap required between the two replicates was increased and the proportion of still-overlapping footprints plotted. In DNase1-seq data, the pipeline optimized to reproduce intermediate alignment and OCR files produces more reproducible footprints than AUC-optimized or default pipelines. **c** The relationship between footprint reproducibility and ChIP-seq AUC was measured. Each pipeline’s proportion of footprints that overlap with the footprints of its replicate (at 1% overlap required) is plotted against that pipeline’s mean AUC. Pipelines with the highest AUC for each data type are marked with arrows

**Fig. 6:**
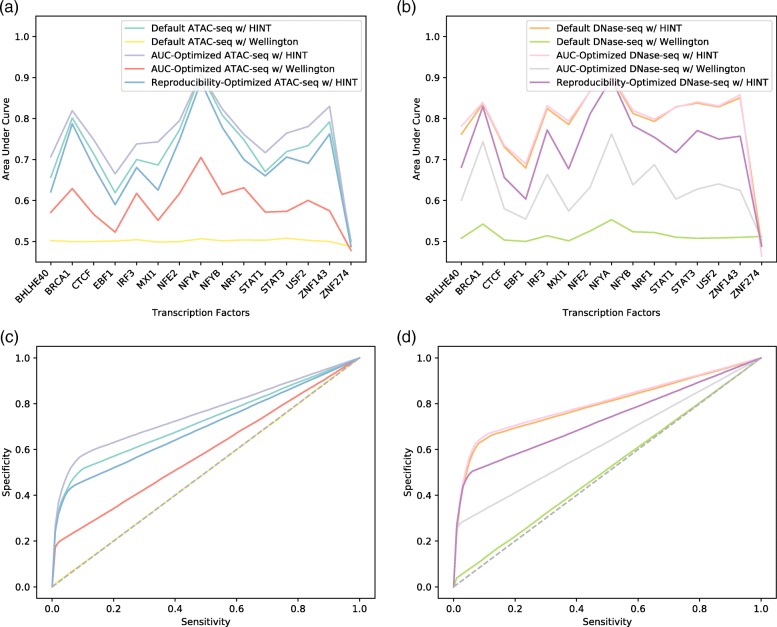
Impact of ChIP-seq recovery optimization on ATAC-seq and DNase1-seq footprinting performance. **a** Per-TF ChIP-seq recovery for ATAC-seq pipelines. **b** Per-TF ChIP-seq recovery for DNase1-seq pipelines. **c** ROC curves for ATAC-seq ChIP recovery. **d** ROC curves for DNase1-seq ChIP recovery. There is an imbalance between negative TFBSs and positive TFBSs of approximately 10:1, and footprint algorithms are conservative and tend towards specificity over sensitivity

DNase1-seq and ATAC-seq footprints were evaluated both for their isogenic replicate reproducibility and their ability to recapitulate known transcription factor binding patterns as described by ChIP-seq (Fig. 5). Optimizing for reproducibility decreased the mean AUC of the pipeline relative to a default set of parameters. Reproducibility-optimized mean AUCs above random decreased relative to default HINT pipelines’s mean AUCs by 21 and 13% for DNase- and ATAC-seq, respectively. Put another way, the pipelines selected for maximal biological reproducibility were not optimal for AUC recovery. AUC optimized HINT pipelines improved ChIP recovery AUC by 3 and 15% for DNase1- and ATAC-seq, respectively (Fig. 6).

### ChIP optimized/reproducibility optimized pipelines

Optimized Snakemake pipelines for ATAC and DNase1 footprinting are available for download at the Chiorini Lab github page (www.github.com/ChioriniLab).

## Discussion

Chromatin accessibility profiling and transcription factor footprinting analyses represent an exciting opportunity to glean new insights on fundamental principles of gene regulation and expression. Herein, we present an optimized and integrated pipeline for the robust analysis of chromatin accessibility data that processes all steps from raw FASTQ input to production of a final, optimized gene regulatory network. These pipelines are freely accessible, and our approaches and data are available for additional optimization as new algorithms for footprinting analysis become available.

### Diminishing return on AUC at 60-80 M reads

Recovery of transcription factor footprints within the ATAC/DNase sequencing signal requires deep sequencing to identify the protein bound region of DNA protected from enzymatic cleavage. We identified a linear relationship between read depth and footprints quantity for both the DNase and ATAC-seq data. Our results indicate that even 200 million reads is insufficient to exhaust the biological information gathered from footprinting, an observation also noted in Barozzi et al. [14]. Deeper sequencing and downsampling will be necessary to determine if there is a natural plateau to the number of footprints that can be detected across the genome.

We noted diminishing returns in ChIP recovery as sequencing depth increases over 60–80 million reads for both ATAC and DNase-seq. Mean AUC at the 100 million read depth was within 80% of the 200 million read depth AUCs for both data types, and mean AUC at the 160 million read depth was within 95% of the 200 million read depth mean AUC for both data types. This plateau was observed in all individual TF without obvious differences in DNA binding kinetics between TF. One explanation is that all TF share the same kinetic properties, or footprints detected at higher read ranges describe transient TF binding that is not detectable in a ChIP-seq assay, or that the footprints detected at higher read depths have diminishing accuracy. Regardless, our data supports that 100 million reads is a defensible read depth for a sequencing run and 160 million reads is an optimal read depth.

### Reproducibility vs. transcription factor ChIP recovery

Utilizing Picard to remove putative PCR duplicates improves biological reproducibility scores between biological (DNase1-seq) and technical (ATAC-seq) replicates. Similarly, smaller peak sizes of open chromatin regions (OCRs) lead to reproducibility improvements. Because of these small peak sizes, there is no reproducibility-optimized Wellington pipeline, as Wellington requires the sizes of shoulder and footprint sequences to sum to a value greater than 50 base pairs. Picard PCR duplicate removal is used in some AUC-optimized pipelines, whereas small peak sizes are not used in any AUC-optimized pipelines.

PCR duplicate removal takes out identically-ended sequencing reads (reads that look like the result of PCR bias). For example, in a chromatin accessibility assay performed on a population of 50,000 cells, it is expected that the enzyme cuts the same base pair position in multiple genomes at some unknown rate. Pipelines that used PCR duplicate removal exhibited as much as a 50% reduction in the number of footprints found relative to their untreated counterpart pipelines. In our data, TF footprinting detection by Wellington was negatively impacted by PCR duplicate removal, with a mean reduction of 84% of the AUC above random. Similarly, larger peak sizes increase the genomic interval in which footprinting is detected while decreasing the degree of biological reproducibility. Lost opportunities to call footprints, in the case of peak size, or lost information to call footprints, in the case of PCR duplicate removal, may explain the apparent contradictions we observed between increased reproducibility and diminished AUC scores for certain pipelines.

We noted that pipelines optimized for reproducibility in alignment and peak calling do not score well on AUC, and vice versa. This tradeoff is explicable by the two argument differences between these pipelines: HOMER peak size and bias correction. High reproducibility was found in samples with small peak sizes, and visual inspection determined those peaks overlapped with only the highest points of alignment, leading to safe and reliable peak assertions. However, as peaks are the only regions in which footprints are searched for, fracturing of open chromatin regions limits footprinting in regulatory regions and can reduce AUC. HINT’s known DNase bias correction increases reproducibility in DNase-seq footprints but reduces mean AUC. This phenomenon is currently inexplicable. Interestingly, none of the evaluated bias correction methods improved ATAC-seq footprint reproducibility or mean AUC.

It is worth noting that overlap of footprints between replicates was higher in DNase1-seq pipelines optimized for reproducibility relative to AUC-optimized or default pipelines, and lower in ATAC-seq pipelines optimized for reproducibility relative to AUC-optimized or default pipelines. It is not clear why this is the case. Using an AUC-optimized pipeline with HINT results in the highest biological information possible gleaned from footprinting.

We also found that AUCs representing ChIP recovery for ATAC-seq data in GM12878 were universally lower than ChIP recovery AUCs from DNase-seq data. Our optimized pipelines for ATAC-seq data used very sensitive alignment but were otherwise identical to optimized pipelines for DNase-seq for each algorithm. It may be the case that algorithms have been optimized on DNase-seq data and modes for using ATAC-seq data are not mature. Neither optimized pipeline using HINT utilized either known DNase1 bias or bias estimated from the data. As footprinting algorithms continue to include ATAC-seq training data, it is very likely that performance on this kind of data will improve.

## Conclusions

Chromatin accessibility profiling assays such as ATAC-seq and DNase1-seq offer the opportunity to rapidly characterize the regulatory state of the genome at a single nucleotide resolution. Significant optimization of molecular protocols has enabled the generation of sequencing libraries in several hours. The remaining computational analysis of next generation sequencing data is the primary obstacle to wide-scale deployment of accessibility profiling assays (e.g., ATAC-seq). To address this obstacle, we have developed an optimized and efficient pipeline for the analysis of ATAC-seq and DNase1-seq data.

Our pipeline was designed to maximize ChIP recovery, the current ‘gold standard’ in transcription factor occupancy assessment. In addition, we performed a computationally intensive grid search across 4560 possible argument combinations to identify the impact of parameter selection on pipeline performance. Our analyses identified the optimal argument combinations for ATAC-seq and DNase1 analysis and these pre-optimized pipelines were translated into Snakemake pipelines for routine and reproducible deployment to supercomputing clusters. Each iteration of these analyses required approximately 11.5 CPU years to reach completion and are being made freely available at the Chiorini Lab Github to avoid duplication of effort within the scientific community. Additionally, the raw datasets and code for pipeline validation have been made publically available allowing investigators to easily profile, and rapidly improve, future pipelines and algorithms for transcription factor footprinting.

Genome wide chromatin accessibility mapping offers a unique window into the regulatory state of the cell by allowing an investigator to rapidly map cellular genomic states. We anticipate continued development in algorithms and molecular protocols that will improve the accuracy and efficiency of gene regulatory network inference from ATAC/DNase sequencing. As chromatin profiling is a valuable tool to characterize the networks and TF binding events that define a cell’s operation, we expect that chromatin profiling assays will continue to see use in cancer genomics, cell engineering, and monogenic diseases cases evading exomic diagnosis. The optimized analysis pipelines and quantitative profiling metrics for construction of ATAC/DNase-seq networks provided here contribute to the scientific community by providing an easily accessed platform for analysis and pipeline engineering.

## Methods

### Pipeline

**Fig. 7:**
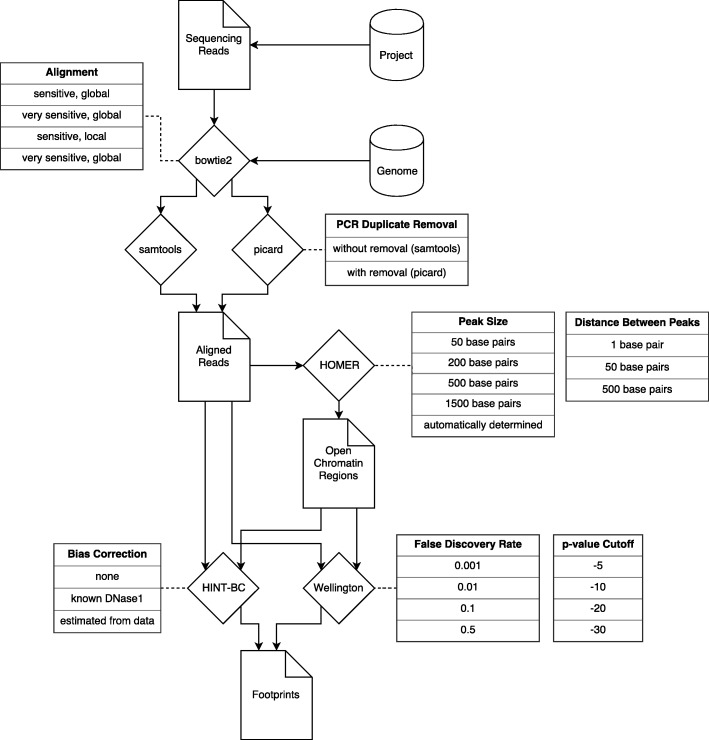
Flowchart of the pipeline. Dog-eared squares are files, diamonds are software packages, tables represent the arguments passed to the pipeline

The structure of our footprinting analysis pipeline is presented in Fig. 7 and Additional file 1: Table S1. Briefly, the first step in the pipeline was to perform quality control on the FASTQ file of raw reads from the sequencing instrument using FastQC (http://www.bioinformatics.babraham.ac.uk/projects/fastqc). For ATAC-seq, FastQC adds additional information by identifying the tags remaining in the read from Tn5 tagmentation. Removal of tagmentation tags was accomplished via truncation of 20 bp of signal from the 5′ end of the transcript using Trimmomatic [16]. For the ATAC-seq data used to validate the pipeline, the entire read was trimmed to 20 bp to minimize the number of reads resulting from PCR chimerism (Additional file 2: Figure S1 and Additional file 3: Figure S2).

Following quality control, the second step is to align reads to the genome using Bowtie2 [17]. A series of alignment arguments are passed to the pipeline. These first two arguments specify if Bowtie2 is to align reads globally or locally as well as sensitively or very sensitively. A global alignment subtracts overhangs in the sequenced reads from the alignment score, whereas a local does not. “Sensitive” and “very sensitive” are modes of alignment used by Bowtie2. Running Bowtie2 on “very sensitive” causes the program to attempt more potential alignments than “sensitive” before discarding or aligning a read.

In the third step, aligned reads are converted to a binary format using (as the third argument) either SAMtools [18] or the Broad Institute’s Picard package (http://broadinstitute.github.io/picard). These tools are then used to sort and index the aligned reads. If Picard is used to convert the reads to binary format, the aligned reads are also queued for PCR duplicate removal via the Picard MarkDuplicates function. PCR duplicates are identical copies of reads which can introduce PCR bias to the TF footprinting analysis by over-representing a small number of highly duplicated genomic regions. In genomic footprinting, PCR duplicate removal may lower bias or, alternatively, discard biological information.

In the fourth step, the aligned reads are analyzed by the HOMER [19] findpeaks function to identify open chromatin regions (OCRs). OCRs are operationally described as intervals of the genome with higher aligned read density than background. Functionally, OCRs are therefore regions of the genome with low nucleosome occupancy that demonstrate higher enzymatic accessibility and read signal. Following OCR identification, the computationally expensive HINT [21] and Wellington [22] footprinting algorithms are targeted to these areas of the genome HOMER identified as peaks to search for TF footprints using alignment information. The “peak height” of an OCR corresponds to the number of reads that align to that region and is a metric that quantitatively describes the chromatin accessibility of a genomic interval. The two arguments passed to HOMER to process aligned reads are the OCR’s sizes, which are the size in base pairs of the regions that HOMER uses to scan across the genome (and are thus the lower limit on the length of a peak); and the minimum distance allowed between OCRs. These are the fourth and fifth arguments in the tested pipelines.

The final step of the analysis pipeline is to pass the aligned reads and OCRs to a TF footprinting identification algorithm. We evaluated two footprinting algorithms, Wellington and HINT. Wellington utilizes a beta-binomial distribution to estimate footprints and leverages the observation that enzymatically accessible genomic DNA in the nucleus results in strand-specific increases in enzyme activity around transcription factor footprints. Wellington requires two arguments: a *p*-value cutoff and a desired false discovery rate. HINT is a hidden Markov model based footprinting algorithm that incorporates bias correction for known DNase1 digestion biases. Usefully, HINT also incorporates functions that enable de novo estimation of enzymatic bias from input data. The only parameter that can be passed to HINT is the mode of bias correction. Both Wellington and HINT return genomic intervals of predicted protein binding. These proposed protein binding intervals are then intersected on the genome with transcription factor position weight matrices to generate predicted transcription factor binding intervals and a final gene regulatory network.

### Pipeline validation metrics

The data used to validate this pipeline came from GM12878, a lymphoblastoid cell line. DNase1-seq data used to validate the pipeline is ENCODE data [13] from the Stamatoyannopolous and Crawford labs downloadable at *https://www.encodeproject.org/experiments/ENCSR000EJD/* and *https://www.encodeproject.org/experiments/ENCSR000EMT/**,* respectively*.* ATAC-seq data used to validate the pipeline was acquired from Buenrostro et al. [6] via the Gene Expression Omnibus repository GSE47753. Biological reproducibility was assessed by assembling at least two independently sampled datasets for each source (ATAC-seq and DNase1-seq). All the ATAC-seq data was produced within the Buenrostro Lab, whereas the DNase1-seq data is replicated across the Stamatoyannopoulos lab and Crawford labs, potentially impacting observed reproducibility due to technical and biological variance.

Three metrics of data reproducibility were assayed across the pipeline. The first metric was the correlation of sequence read alignments. Under this metric, the genome is divided into 10 kilobase bins and the number of reads in each bin is compared between replicates for each bin. A summary Pearson correlation coefficient is then calculated, with higher Pearson correlation coefficients between genome bin sets suggestive of similar patterns of aligned reads across the genome.

The second metric of reproducibility quantitatively assesses OCR reproducibility between replicates. Peaks are averaged across 10 kilobase bins and compared between replicates to produce a Pearson correlation coefficient that summarizes the reproducibility of the assay to detect OCRs across multiple replicates.

The final reproducibility metric assayed was the percentage of TF footprints detected as present and overlapping in two biological replicates. This metric assesses the ability of the assay to detect biologically reproducible TF binding events.

In addition to the three metrics of reproducibility, we assessed the ability of chromatin accessibility based data to recover transcription factor binding by comparing identified footprints to the ‘gold standard’ of ChIP-seq for each footprint. Transcription factor ChIP data for BHLHE40, BRCA1, CTCF, EBF1, IRF3, MXI1, NFE2, NFYA, NFYB, NRF1, STAT1, STAT3, USF2, ZNF143 and ZNF274 was downloaded from ENCODE. This data was compared to footprint coverage of the genome at all positions matching that transcription factor’s binding motif to assess the ability of transcription factor footprinting to recover known ChIP-seq binding information.

#### Software

Software used in the pipeline includes FastQC (http://www.bioinformatics.babraham.ac.uk/projects/fastqc), seqtk (https://github.com/lh3/seqtk), Bowtie2 v.2.2.9 [17], SAMtools v.1.3.1 [18], HOMER v.4.8.2 [19], BEDtools v.2.25.0 [23], BEDops v.2.4.26 [24], pyDNase v.0.2.4 [22], R v.3.4, deepTools v.2.2.4 [25], Picard v.2.9.2, UCSC tools v.344 [26], RGT v.0.9.9 [20, 21], and Trimmomatic v.0.36 [16]. All code was translated to the Snakemake [27] pipeline management platform to streamline scientific supercomputing deployment and maximize data reproducibility. This work utilized the computational resources of the NIH HPC Biowulf cluster. (http://hpc.nih.gov).

These software packages were selected based on the original footprinting tutorial from Piper and found at https://pythonhosted.org/pyDNase/tutorial.html. The tested pipelines use software used both in that tutorial and in the Hardison ATAC-seq pipeline found at https://www.encodeproject.org/pipelines/ENCPL035XIO/.

#### Downsampling

ATAC-seq and DNase-seq data was downsampled using seqtk (https://github.com/lh3/seqtk) to 20, 40, 60, 80, 100, 120, 140, 160 and 200 million reads. Random seeds for seqtk were set to the same value as the number of reads being downsampled to and three permutations of the data were iteratively sampled. Downsampled samples were used to assess the impact of read number on the total number of transcription factor footprints detected and AUC recovery.

#### Trimming

ATAC-seq fastq reads were trimmed using Trimmomatic [16] to remove Tn5 tagmentation adapters. The final length of the trimmed determined to be mappable without a high expected rate of random alignment was 20 and Trimmomatic was called with the arguments HEADCROP:20 CROP:20.

#### Alignment

Alignment was performed with Bowtie2 [17]. Alignments were performed using the --sensitive (default), −-sensitive-local, −-very-sensitive, and --very-sensitive-local pre-configured alignment parameters. Default alignments are global, requiring the entire read to align to the genome.

To process alignments for downstream analysis, SAMtools [18] was used to process reads without the removal of PCR duplicates. The impact of PCR duplicate removal was assessed via the use of Picard to process reads and remove PCR duplicates. The SAMtools processes used were view, sort and index, while the Picard processes used were SortSam, MarkDuplicates and BuildBamIndex.

#### Open chromatin regions

Open chromatin regions were identified from alignment files using HOMER [19] makeTagDirectory, findPeaks and pos2BED functions, as well as BEDtools [23] sort and merge functions. These bedfiles representing open chromatin regions were analyzed by the footprint detection algorithms, HINT [21] and Wellington [22].

#### Reproducibility metrics

To compute reproducibility in alignment space, deepTools [25] multiBamSummary and plotCorrelation were used to summarize the alignments in genomic bins of size 10 kb across the hg19 genome and compare these alignments between two biological replicates. High Pearson correlation coefficients describe pipelines in which alignments are mapping to the same genomic bins between two replicates.

To compute reproducibility in open chromatin region calling across the genome, deepTools’ multiBigwigSummary and plotCorrelation were used to quantify the similarity in open chromatin calling between replicates using 10 kb bins. The average score for open chromatin was calculated and compared between bins. High Pearson correlation coefficients are assigned to pipelines in which both replicates have open chromatin regions in the same genomic bins.

#### AUC evaluation

To produce PWM hits for individual proteins, the cisBP [28] database was mapped to the human hg19 genome with FIMO using a *p*-value cutoff of 1E-04 [29]. Transcription factor binding was assessed via analysis of ChIP-seq data for GM12878 acquired from the ENCODE [13] consortium from data accessions beginning in ENCSR000D and ending in NN, ZS, ZN, ZU, ZO, ZM, ZI, NM, ZY, ZX, ZJ, ZQ, ZL, ZV and YW.

The ability of the pipeline to recapitulate known transcription factor binding events was assessed by comparison of the TF footprint predictions with known TF binding events from ChIP-seq data. Footprints overlapping known TF binding intervals via ChIP-seq were assessed as true positives, while footprints without overlapping ChIP-seq peaks were labeled as false positives. ChIP-supported protein binding sites that did not overlap a footprint were considered false negatives, and ChIP negative, footprint negative binding sites were considered to be true negatives. Predictions from footprints were ordered by score from Wellington or HINT, and binding site predictions that didn’t overlap with predictions from footprints were ordered randomly. Receiver operating characteristic (ROC) curves were produced from these predictions and the area under curve (AUC) for each transcription factor was calculated. Across all of the transcription factors tested, there were on average 10 negative TF binding sites for each positive TF binding site.

## Additional files

Additional file 1: **Table S1.** Parameters passed to each pipeline. Default, AUC-optimized and reproducibility-optimized pipelines for ATAC-seq and DNase1-seq are shown using each footprinting algorithm. Parameters for each pipeline are listed. These parameters correspond to Fig. 7, and these pipelines can be found at github.com/ChioriniLab. (PDF 182 kb)

Additional file 2: **Figure S1.** FastQC Reveals Tn5 Tags and PCR Chimerism. Non-random distributions of the first ~ 10 bases after tagmentation correspond to the Tn5 tagmentation after library primers are removed. This non-random region at the beginning of the read is characteristics of ATAC-seq data. In the ATAC-seq sample from Buenrostro, a second non-random region can be seen, suggesting PCR chimerism. (PDF 57 kb)

Additional file 3: **Figure S2.** Trimming reads improves alignment of the GM12878 ATAC-seq reads. Tn5 transposase attaches mosaic end (ME) tags that need to be trimmed from the 5′ end of the read. Additionally, however, trimming low-quality base pairs from the 3′ end of the ATAC-seq reads so that all reads had the same length improved alignment to the genome (shown in green). With a 3 billion base pair genome, the chance that a sequence of a certain length will align randomly is high for sequences shorter than 17 base pairs. To minimize random alignment while removing low-quality base pairs for this ATAC-seq data, we trimmed the reads to a final length of 20 base pairs. (PDF 13 kb)

Additional file 4: **Figure S3.** Visual inspection reveals consistent overlap between HOMER peaks and OCRs at peak size of 200. **(a)** Read coverage for DNase1-seq (top) and ATAC-seq (bottom) shown underneath open chromatin regions called by HOMER at peak sizes ranging from 10 base pairs to 2000 base pairs. Peak reproducibility between replicates was shown to be higher with lower peak sizes. Visualized using the Broad Institute’s Integrative Genomics Viewer (software.broadinstitute.org/software/igv/). **(b,c,d)** Metrics of reproducibility and biological information plotted against the HOMER argument minDist for all pipelines. Pipelines could have a minDist of 0, 50 or 500, and these values had no effect on correlation between replicates or recapitulation of known ChIP-seq. (PDF 1272 kb)

## Abbreviations

ATAC-seq: Assay for Transposase-Accessible Chromatin using sequencing
AUC: Area under the curve
ChIP-seq: Chromatin immunoprecipitation
DNase1-seq: DNase1 hypersenstivity profiling
GRN: Gene regulatory network
NGS: Next-generation sequencing
OCR: Open chromatin region
PCR: Polymerase chain reaction
RNAP: RNA polymerase
ROC: Receiver operating characteristic (curve)
TF: Transcription factor
