# Correction to: ATAC2GRN: optimized ATAC-seq and DNase1-seq pipelines for rapid and accurate genome regulatory network inference

**DOI:** 10.1186/s12864-019-5441-7

**Published:** 2019-01-15

**Authors:** Thomas J. F. Pranzatelli, Drew G. Michae, John A. Chiorini

**Affiliations:** National Institute of Dental and Craniofacial Research, National Institutes of Health, 10 Center Drive, Bethesda, MD 20816 USA

**Correction to: BMC Genomics**

**https://doi.org/10.1186/s12864-018-4943-z**

Following the publication of this article [1], the authors informed us of the following typographical errors in the Results section (the changes are marked in bold).

1. The sub-heading “Footprints increase linearly with reads – ChIP-seq recovery yields diminishing returns with read depths greater than 60 million readz” should be “Footprints increase linearly with **reads** – ChIP-seq recovery yields diminishing returns with read depths greater than 60 million reads”.
2. The sentence “ROC AUC (area under the repeater operating characteristic curve), a measure of a predictor’s ability to predict true positives, increased non-linearly as read depths increased (Fig. 2b)” should read “ROC AUC (**area under the receiver operating characteristic curve**), a measure of a predictor’s ability to predict true positives, increased non-linearly as read depths increased (Fig. 2b).”
