# Supplementary material for: ATAC2GRN: optimized ATAC-seq and DNase1-seq pipelines for rapid and accurate genome regulatory network inference
Source: BMC Genomics. 2018 Jul 31;19:563. doi: 10.1186/s12864-018-4943-z (PMC6069842; doi:10.1186/s12864-018-4943-z)
Supplement: Supplementary file 1 — Table S1. Parameters passed to each pipeline. Default, AUC-optimized and reproducibility-optimized pipelines for ATAC-seq and DNase1-seq are shown using each footprinting algorithm. Parameters for each pipeline are listed. These parameters correspond to Fig. 7, and these pipelines can be found at github.com/ChioriniLab. (PDF 182 kb) [file 12864_2018_4943_MOESM1_ESM.pdf]

Pipeline	Alignment	PCR Duplicate Removal	HOMER Peak Size	HOMER minDist	p-value	FDR	Bias Correction
Default ATAC-seq w/ HINT	sensitive global	without	500	50	N/A	N/A	none
Default ATAC-seq w/ Wellington	sensitive global	without	500	50	-20	0.01	N/A
Default DNase1-seq w/ HINT	sensitive global	without	500	50	N/A	N/A	none
Default DNase1-seq w/ Wellington	sensitive global	without	500	50	-20	0.01	N/A
AUC-Optimized ATAC-seq w/ HINT	very sensitive global	with	500	50	N/A	N/A	none
AUC-Optimized ATAC-seq w/ Wellington	very sensitive global	without	200	50	0.1	-5	N/A
AUC-Optimized DNase1-seq w/ HINT	sensitive global	with	500	50	N/A	N/A	none
AUC-Optimized DNase1-seq w/ Wellington	very sensitive local	without	200	50	0.1	-5	N/A
Reproducibility-Optimized ATAC-seq w/ HINT	sensitive global	with	50	50	N/A	N/A	DNase1
Reproducibility-Optimized DNase1-seq w/ HINT	sensitive global	with	50	50	N/A	N/A	DNase1
