# Supplementary figures and images for: ATAC2GRN: optimized ATAC-seq and DNase1-seq pipelines for rapid and accurate genome regulatory network inference
Source: BMC Genomics. 2018 Jul 31;19:563. doi: 10.1186/s12864-018-4943-z (PMC6069842; doi:10.1186/s12864-018-4943-z)

## Per base sequence content

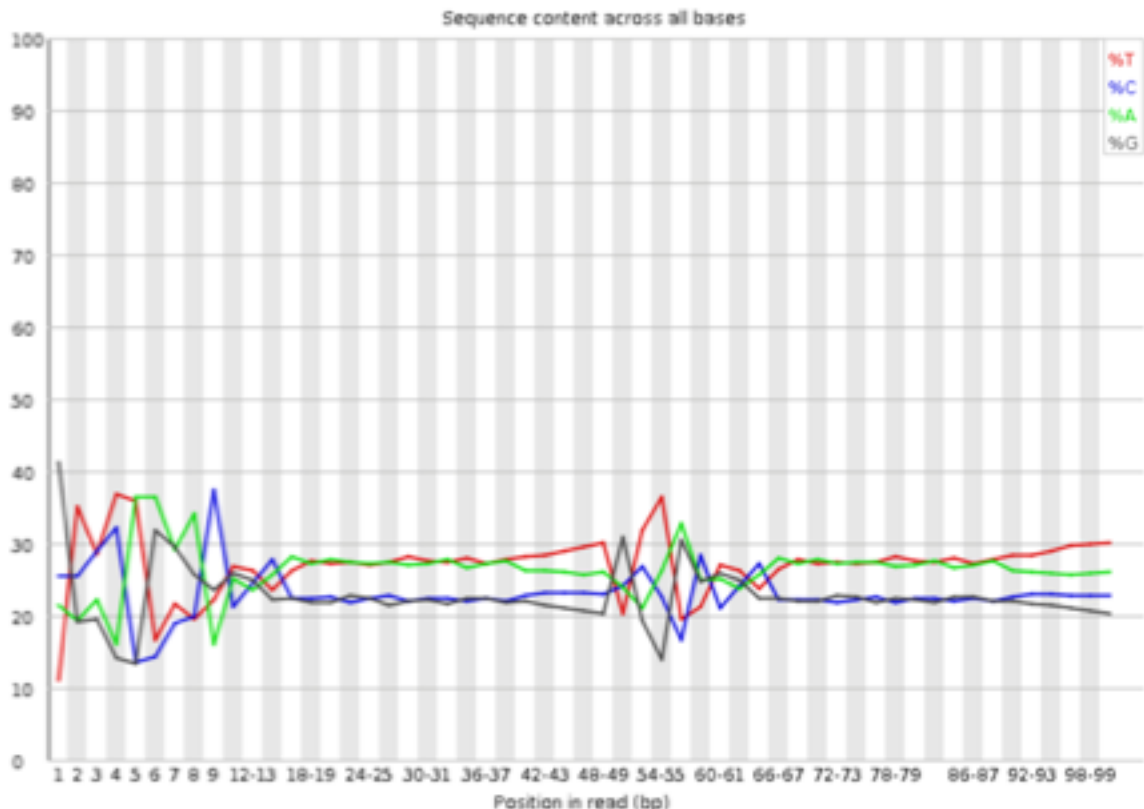

Supplement: Supplementary file 2 — Figure S1. FastQC Reveals Tn5 Tags and PCR Chimerism. Non-random distributions of the first ~ 10 bases after tagmentation correspond to the Tn5 tagmentation after library primers are removed. This non-random region at the beginning of the read is characteristics of ATAC-seq data. In the ATAC-seq sample from Buenrostro, a second non-random region can be seen, suggesting PCR chimerism. (PDF 57 kb) [file 12864_2018_4943_MOESM2_ESM.pdf]

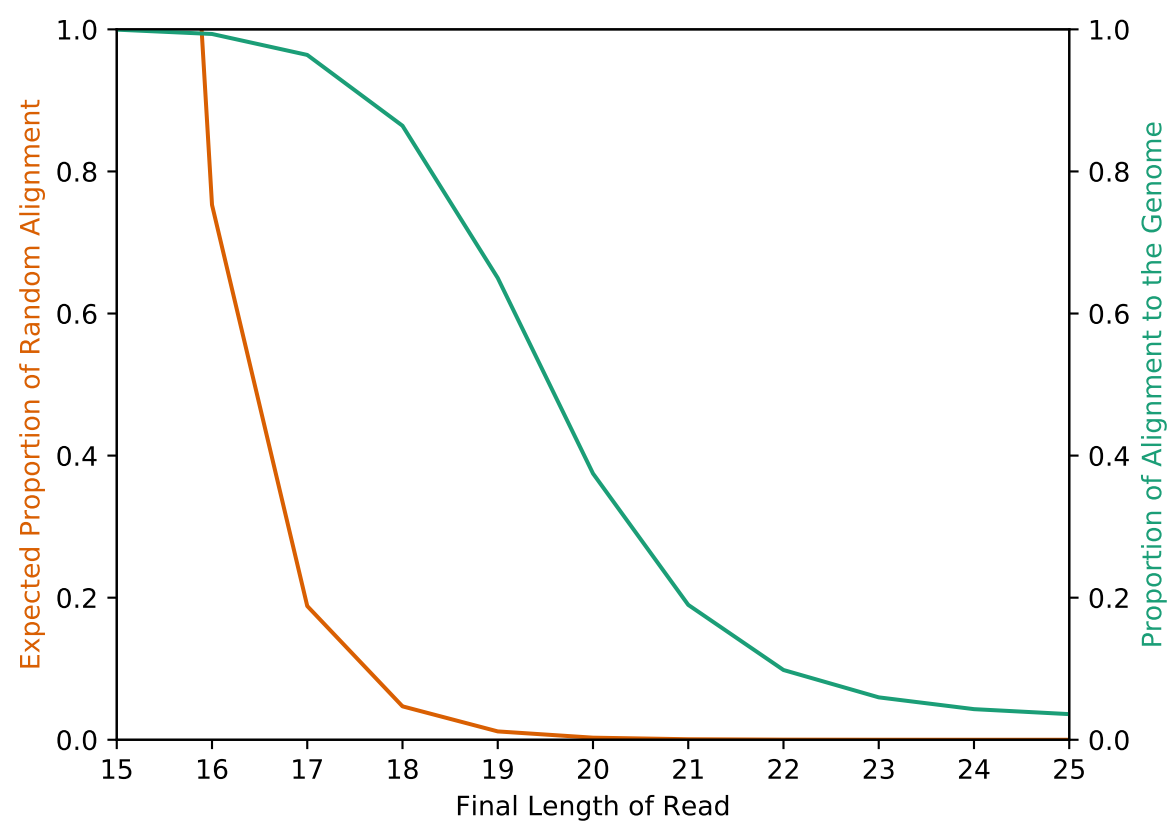

Supplement: Supplementary file 3 — Figure S2. Trimming reads improves alignment of the GM12878 ATAC-seq reads. Tn5 transposase attaches mosaic end (ME) tags that need to be trimmed from the 5′ end of the read. Additionally, however, trimming low-quality base pairs from the 3′ end of the ATAC-seq reads so that all reads had the same length improved alignment to the genome (shown in green). With a 3 billion base pair genome, the chance that a sequence of a certain length will align randomly is high for sequences shorter than 17 base pairs. To minimize random alignment while removing low-quality base pairs for this ATAC-seq data, we trimmed the reads to a final length of 20 base pairs. (PDF 13 kb) [file 12864_2018_4943_MOESM3_ESM.pdf]

a)

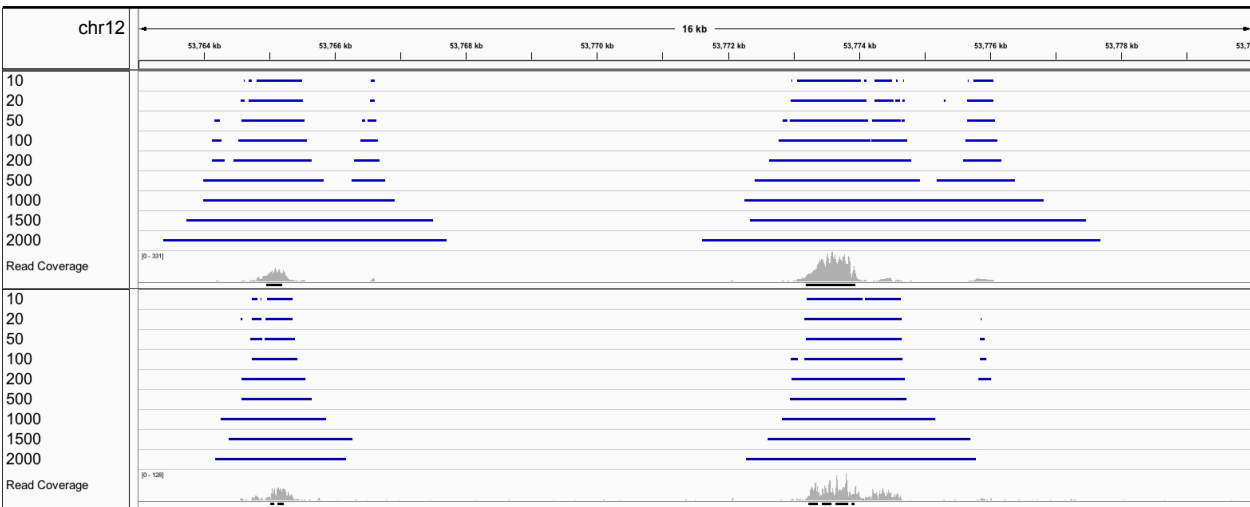

b)

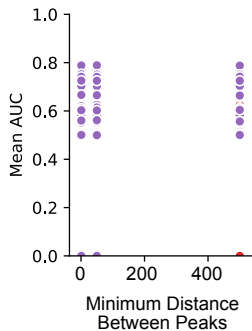

c)

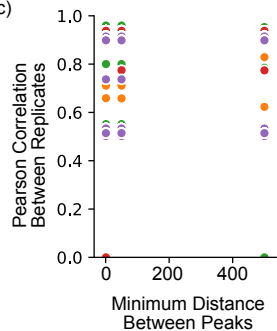

d)

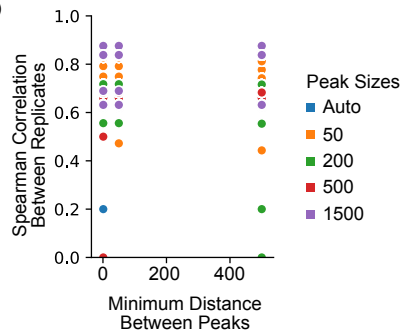

Supplement: Supplementary file 4 — Figure S3. Visual inspection reveals consistent overlap between HOMER peaks and OCRs at peak size of 200. (a) Read coverage for DNase1-seq (top) and ATAC-seq (bottom) shown underneath open chromatin regions called by HOMER at peak sizes ranging from 10 base pairs to 2000 base pairs. Peak reproducibility between replicates was shown to be higher with lower peak sizes. Visualized using the Broad Institute’s Integrative Genomics Viewer (software.broadinstitute.org/software/igv/). (b,c,d) Metrics of reproducibility and biological information plotted against the HOMER argument minDist for all pipelines. Pipelines could have a minDist of 0, 50 or 500, and these values had no effect on correlation between replicates or recapitulation of known ChIP-seq. (PDF 1272 kb) [file 12864_2018_4943_MOESM4_ESM.pdf]
